# Short-term effects of wildfire on soil arthropods in a semi-arid grassland on the Loess Plateau

**DOI:** 10.3389/fmicb.2022.989351

**Published:** 2022-10-21

**Authors:** Xi Yang, Ren-Tao Liu, Ming-An Shao, Xiao-Rong Wei, Tong-Chuan Li, Ming-Yu Chen, Zhi-Yong Li, Yun-Chao Dai, Miao Gan

**Affiliations:** ^1^State Key Laboratory of Soil Erosion and Dryland Farming on the Loess Plateau, Institute of Soil and Water Conservation, Chinese Academy of Sciences, Yangling, China; ^2^College of Resources and Environment, Northwest A&F University, Yangling, China; ^3^Breeding Base for State Key Laboratory of Land Degradation and Ecological Restoration in Northwestern China, Ningxia University, Yinchuan, China; ^4^Key Laboratory of Restoration and Reconstruction of Degraded Ecosystems in Northwestern China of Ministry of Education, Ningxia University, Yinchuan, China

**Keywords:** wildfire, recovery, soil arthropods, seasonal variations, Loess Plateau

## Abstract

Fires lead to dramatic shifts in ecosystems and have a large impact on the biota. Soil organisms, especially soil fauna, are often used as indicators of environmental change. At present, minimal attention has been paid to using soil fauna as an indicator of environmental change after a fire. Here, a field survey of burnt herbaceous vegetation in semi-arid areas was conducted to determine the response of soil arthropods to fire and their short–term recovery after fire. Overall, the abundance and biomass of soil arthropods was more sensitive to fire than the number of groups. The number of soil arthropod groups, especially the dominant groups (mites and springtails), was not significantly affected by wildfires. At the unburned site, soil arthropod abundance showed significant seasonal shifts that may be related to the vegetation properties, temperature, and precipitation caused by seasonal changes. In contrast, soil arthropods at the burnt sites showed a delayed recovery and had only reached 56%–82%, 17%–54%, and 91%–190% of the biomass in the unburnt forest at the 3, 6, and 9 months after the burning event. Our findings of soil arthropod abundance changes in the present study suggest that fire-induced changes in soil and vegetation properties (e.g., AN, LT, and VC) were crucial factors for the changes in soil arthropod abundance in this semi-arid grassland. We conclude that fire disturbance reduces the seasonal sensitivity of soil arthropods by altering their habitat. This study furthers our understanding of wildfire impact recovery by documenting the short-term temporal dynamics of soil arthropods.

## Introduction

Fire is one of the most widespread disturbances worldwide (Coyle et al., 2017), and it plays a critical role in ecosystem patterns and processes (Bowman et al., 2009). As one of the disturbances that trigger “the ecosystem renewal cycle” (Peterson et al., 1998; Bengtsson et al., 2000), fire can alter vegetation dynamics, carbon sequestration, nutrient cycling, climate, and biodiversity (Cook, 1994; Williams et al., 2004; Kelly et al., 2015; Gordon et al., 2018; Lasslop et al., 2019). Over the next century, the increased risk of fires worldwide is likely to pose a greater threat to global ecosystems (Pellegrini et al., 2018; Stevens-Rumann et al., 2018). The effects of fire on biogeochemical cycles are well documented (Pellegrini et al., 2018; Dove et al., 2020), but the consequences for soil organisms, especially soil fauna, are seldom addressed.

The wide variety and large number of soil fauna are important components of terrestrial ecosystems (Decaёns et al., 2006; Yang et al., 2021) and are sensitive to habitat changes (Ayres et al., 2009; Carrillo et al., 2011). Fire is a common disturbance that significantly impacts soil fauna (Nick, 2003; Louzada et al., 2010). This may be attributed to the fact that fires can return biological elements in tree biomass to the soil in the form of ash (Zaitsev et al., 2014) and cause a large loss of organic matter and macro-elements (Bormann et al., 2008). In addition, fires tend to burn most of the aboveground vegetation and organic layers and change the hydrothermal conditions of the soil (Zaitsev et al., 2014). The remaining organic matter is mostly well-decomposed and offers an inferior substrate for decomposers than unburnt soil and litter (Gongalsky et al., 2012). Therefore, the development of post-fire soil biota communities is determined by these factors, and the soil food web structure is completely different from that before a fire (Huhta, 1971; Holliday, 1991; Gongalsky and Persson, 2013).

Whether prescribed or naturally occurring, fire can affect this soil biological community in a direct (by killing or injuring organisms) or indirect manner (by reducing habitat availability and food resources; Certini, 2005; Coyle et al., 2017). However, the characteristics of the fire and the habits of the soil organisms themselves are the main factors that determine the intensity of fire disturbance (Coyle et al., 2017). Intensive fires damage the litter layer, the soil organic layer, and all inhabiting animals (Phillips et al., 2000; Malmström, 2012). The survival rate of animals after a moderate fire is 42%–62% (Gongalsky et al., 2012). In addition, different animal species respond differently to fires. For example, compared with endogeic organisms, epigeic organisms are more susceptible to fire (Coleman and Rieske, 2006). Extreme heat during fires or the loss of substrate/habitat due to burning can kill epigeic fauna that cannot penetrate the soil or escape quickly (Coyle et al., 2017). However, endogeic organisms often utilize more (and deeper) portions of the soil profile (e.g., ants) and are thus less influenced by extreme heat during fires (Silveira et al., 2013; Andersen et al., 2014). Carabids and spiders survive fires by burrowing, flying, or finding refuges (McCullough et al., 1998). Earthworms survive fire by descending into the soil (Leonard, 1977). The survival of ground beetles relies on their thick cuticle (Wikars and Schimmel, 2001). However, a few taxa, mostly beetles, have also been considered pyrophilic and have preferred recently burned habitats (Wikars, 1997; Buddle et al., 2006; Gandhi et al., 2008).

The post-fire recovery of soil faunal communities depends on abiotic and organism traits (Gongalsky et al., 2012). Organism traits have two main aspects: immigration from the unburnt area and the remaining soil fauna or eggs in the burned area (Gongalsky et al., 2012). The abiotic factors affecting recovery ability mainly include fire severity, topography, fire season, fire heterogeneity, and weather conditions (Hellberg et al., 2004; Lavoie and Mack, 2012). Post-fire recovery is also influenced by the properties of the plant community and the edaphic conditions before the fire (Zaitsev et al., 2016). In addition, the species composition, coverage, and litter production of the plant community vary seasonally, changing the microhabitats available to the soil fauna (Wu et al., 2014). Therefore, seasonal changes in the plant community can directly or indirectly influence soil fauna communities (Wu et al., 2014). Moreover, seasonal changes in temperature and precipitation are closely related to seasonal changes in the soil fauna (Irmler, 2006). However, it is unclear which of these factors (vegetation, temperature, precipitation, and other factors) is the most important in determining the seasonal dynamics of soil fauna following fire.

Wildfire is one of the most important natural disturbance processes in global ecosystems, and it has long-term and extensive effects on most terrestrial ecosystems (Yue et al., 2020). Approximately 35–47 million hectares of forested areas are affected by fires globally each year (Mouillot and Field, 2005). At the same time, grassland ecosystems, which account for 40.5% of the Earth’s ice-free terrestrial surface, are also often threatened by fires (White et al., 2000). It has been estimated that about 80% of the world’s wildfires occur annually in grass-dominated communities (Mouillot and Field, 2005). Although research on the effects of forest fires on soil fauna was initiated many decades ago (Heyward and Tissot, 1936), studies on the effects of fires on grassland soil organisms in semi-arid areas are still scarce, especially the short-term dynamics of soil fauna after fires. Therefore, in this study, soil arthropods at different stages of herbaceous vegetation restoration after fire were selected as research objects, and the recovery dynamics, distribution patterns, and main factors affecting the recovery of soil arthropods after fire were determined by investigating the diversity of soil arthropods after fire. We hypothesized that (1) fires reduce the groups and abundance of soil arthropods mainly through direct killing or injuring; (2) the groups and abundance of soil arthropods continue to increase with recovery time; and (3) vegetation characteristics may be most important in determining the short-term recovery dynamics of soil arthropods following fire.

## Materials and methods

### Study areas

**Figure 1:**
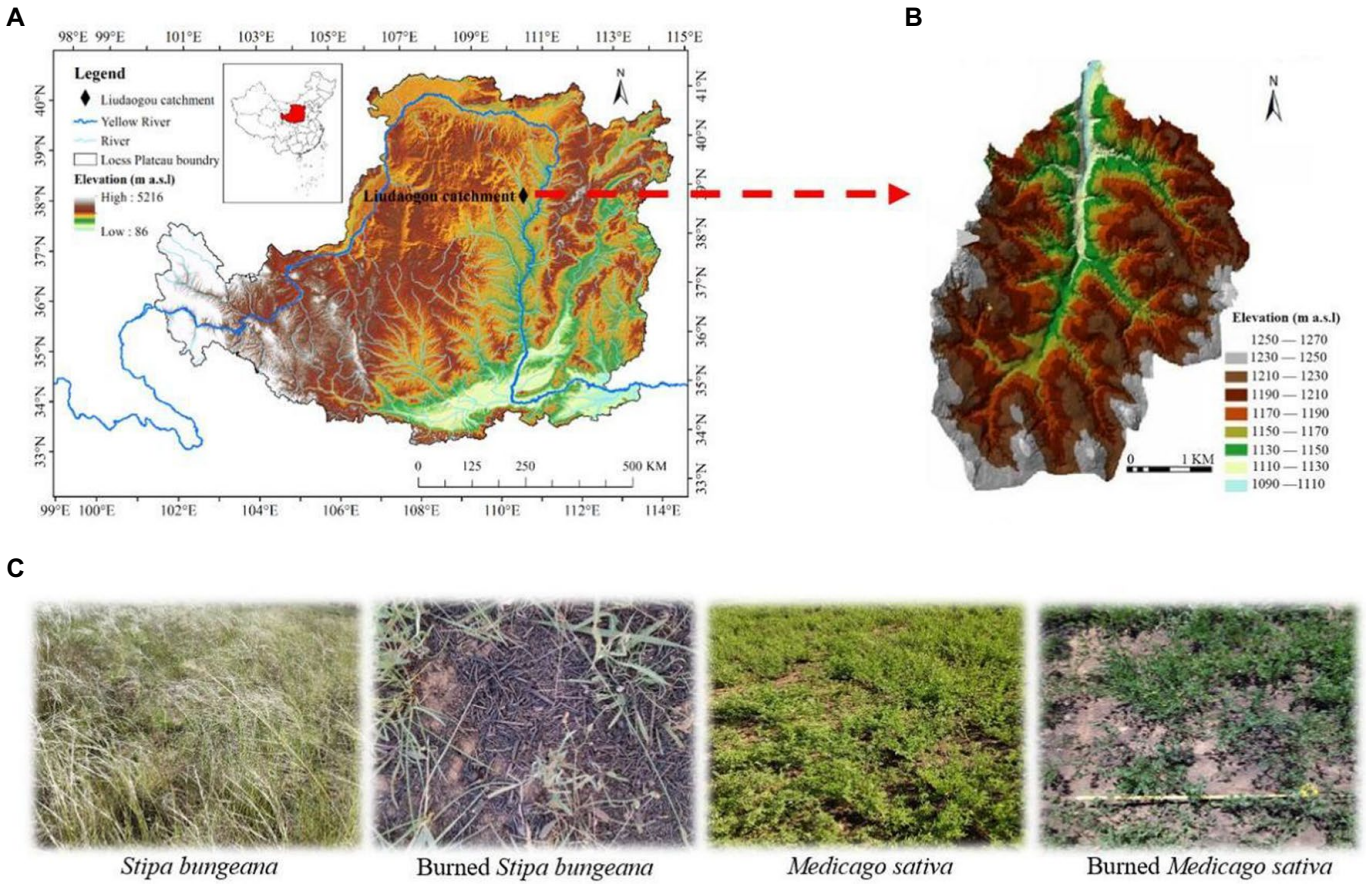
Location of the study site **(A,B)**, and selected habitat type **(C)** in Liudaogou Catchment, Loess Plateau region, China. The map was created in ArcGIS developed by Esri (Environmental Systems Resource Institute, ArcGIS 10.0; www.esri.com). The habitat type **(C)** was photographed in May.

The study was conducted in the Liudaogou catchment (E110°21′–110°23′ N38°46′–38°51′), which is located on the northern Loess Plateau, China (Figures 1A,B). This region has a moderate semiarid climate, with a mean annual aridity index of 1.8 and mean annual potential evapotranspiration of 785 mm. The average precipitation and temperature in spring, summer, autumn, and winter were 72 mm and 11°C, 313 mm and 25°C, 124 mm and 9°C, 8 mm and −5°C, respectively (2009–2018 data from local weather stations). The soil in the study area belongs to eolian loess of Calcaric Regosol (clay, 11%–14%; silt, 30%–45%; sand, 45%–51%; Liu and Shao, 2014). Common vegetation types in this area include *Stipa bungeana* Trin., *Medicago sativa* Linn., *Caragana korshinskii* Kom., *Populus simonii* Carr., *Pinus tabuliformis* Carr., *and Salix matsudana* Koidz.

In February 2019 (winter), approximately 6 ha of the study area was burned during a wildfire. Wildfire studies are reactive and often opportunistic due to the unpredictable nature of wildfires. The study area was found 3 months after the fire (May) under the leadership of local farmers. As judged from the identification of burned plant residues and roots, and from the vegetation distribution in the area before fire, it was determined that the dominant vegetation in the burned area was *Medicago sativa*, *Stipa bungeana*, and *Caragana korshinskii*, and the proportions after measurement were 40%, 50%, and 10%, respectively. The litter and standing litter of *Medicago sativa*, *Stipa bungeana*, and *Caragana korshinskii* in the burned area were charred or consumed. However, their bases were not deeply burned and remained identifiable. Moreover, the charring of the mineral soil was limited to a few millimeters. Small ashes were blown away by wind after the fire, but larger charred vegetation stems were retained. Therefore, according to Ryan’s (2002) fire severity criterion, fire was light. This study focuses on short-term temporal variations because of the relatively rapid recovery of light-burned systems.

### Collection and processing of samples

Two vegetation types with large burn areas (Burned *Medicago sativa* and Burned *Stipa bungeana*) were selected to analyze the short-term effects of wildfires on soil arthropods. Simultaneously, the same vegetation was selected as the control (*Medicago sativa* and *Stipa bungeana*) in the unburned area, 50 m away from the boundary of the burned area. For similar vegetation types, burnt and unburnt habitats must have similar main features, with the occurrence of fire being the only difference (Mantoni et al., 2020). Finally, four habitats were selected: *Stipa bungeana* (SB), burned *Stipa bungeana* (BSB), *Medicago sativa* (MS), and burned *Medicago sativa* (BMS; Figure 1C).

Three permanently marked plots (15 m × 15 m) spaced approximately 10 m apart were established for each of the four habitat types. Five sampling points were selected from each plot. Sampling was conducted in all four habitats in May (spring), August (summer), and November (autumn), 2019. Rainfall may change the surface soil properties and soil fauna characteristics; hence, sampling was timed to ensure that rainfall events did not occur 7 days prior to sampling. A total of 180 samples (4 habitats × 3 plots × 5 sampling points × 3 sampling times) were collected. Soil macroarthropods were sampled using the TSBF methodology (25 cm × 25 cm, 30 cm deep; Anderson and Ingram, 1993) and were hand-sorted, and preserved in 75% alcohol (Zhu and Zhu, 2015). Microarthropods were extracted from each soil sample using a modified Tullgren funnel extractor for 48 h. All obtained arthropods were stored in 75% alcohol and classified into taxa groups based on the *Pictorial Keys to Soil Animals of China* (Yin, 2000).

Soil samples were taken from the plots at the same time as the soil arthropods were collected. The soil samples were thoroughly mixed in each plot, immediately placed into closed plastic bags, stored at low temperature, and transported to the laboratory. The soil water content (SWC), soil bulk density (SBD), soil pH (pH), soil organic carbon (SOC), soil total nitrogen (TN), soil total phosphorus (TP), available phosphorus (AP), ammonia nitrogen (AN), and nitrate nitrogen (NN) were determined using the method described by Yang et al. (2021). Vegetation cover (VC) and litter thickness (LT) were measured and recorded in each plot.

### Statistical analysis

Soil arthropods were separated and converted into abundance (ind. m^−2^) and groups. Data normality of environmental factors was checked using the Kolmogorov–Smirnov test, and variance homogeneity was tested using Levene’s test. Soil arthropod abundance and group numbers among different habitats during the same sampling period were compared using a one-way ANOVA (LSD test, *p* < 0.05). Pearson’s correlation analysis (two-tailed test) was used to analzse the correlation between the main soil arthropods and environmental factors. The effects of vegetation type (S), sampling time (T), wildfire (W), and their three-way interactions on soil properties, arthropods, vegetation coverage, and litter thickness were tested using a three-way ANOVA. Principal component analysis (PCA) was applied to evaluate the effects of habitat and sampling month on soil arthropod composition. Statistical analysis was conducted using SPSS software (Standard released version 22.0, IBM SPSS Statistics Inc., IL., United States).

Detrended correspondence analysis (DCA) was conducted to estimate the gradient length in soil arthropod abundance data. Because of the long gradient length (<4 SD), redundancy analysis (RDA) was chosen to determine the relative contributions of environmental variables to arthropod communities. Soil arthropods were used as dependent variables, whereas soil properties, vegetation coverage, and litter thickness were used as explanatory variables. Groups with abundances less than 0.1% were excluded from analysis. The soil fauna data were log (x + 1) transformed (Zhu and Zhu, 2015). The significance of the first and all axes was evaluated using Monte Carlo tests (499 times, *p* < 0.05). Variance partitioning analysis (VPA) was performed to examine the relative contributions of soil variables (SBD, SWC, SOC, pH, TN, TP, AN, NN, and AP) and vegetation variables (VC and LT) to the variation in community composition. Statistical analyses were performed in R 4.0.3 (“vegan” package).

## Results

### Composition of soil arthropods communities

A total of 16,676 individuals that belonged to 18 groups were obtained from the four habitats during the three sampling periods. The mean abundance of arthropods was 4, 632 individuals m^−2^. The burned *S. bungeana* habitat had the lowest soil arthropod abundance (2,113 individuals m^−2^), but the *M. sativa* habitat had the highest soil arthropod abundance (5,820 individuals m^−2^). Acari (71.72%) and Collembola (10.44%) were the dominant groups. The common groups were Diplura, Coleoptera larvae, Diptera larvae, Isoptera, and Hymenoptera, accounting for 1.02%, 2.46%, 1.99%, 4.18%, and 5.80% of total individuals, respectively (Table 1).

**Table 1:** Soil fauna abundance (mean individuals m^−2^ ± SE) as influenced by the different habitats and sampling time.

Taxa	Burned *Stipa bungeana*			*Stipa bungeana*			Burned *Medicago sativa*			*Medicago sativa*		
	May	August	November	May	August	November	May	August	November	May	August	November
Araneae	30(10)	35(15)	36(15)	24(15)	40(20)	26(15)	44(25)	445(25)	50(20)	30(10)	65(35)	36(25)
Acari	325(80)	340(90)	895(21)	526(215)	7,954(2735)	1,465(255)	2,480(470)	3,070(930)	8,570(307)	1,626(365)	8,086(1095)	4,540(1630)
Geophilomorpha	0	5(5)	0	0	6(5)	0	0	6(6)	0	0	0	0
Lithobiomorpha	0	0	0	0	0	0	0	10(10)	0	0	0	0
Symphyla	0	0	0	0	0	0	0	0	0	0	7(5)	0
Protura	0	0	5(5)	0	0	0	0	25(5)	0	0	0	0
Collembola	600(120)	845(240)	940(150)	226(95)	985(125)	355(65)	106(25)	442(13)	166(65)	125(35)	820(190)	196(75)
Diplura	9(10)	16(15)	0	30(11)	130(20)	0	16(5)	72(5)	0	40(20)	256(85)	0
Isoptera	165(65)	56(35)	356(18)	215(65)	276(105)	460(150)	36(15)	80(5)	75(30)	55(25)	445(135)	110(50)
Hemiptera	5(5)	10(10)	5(5)	6(5)	16(5)	10(10)	10(10)	16(6)	35(15)	5(5)	15(5)	4(2)
Homoptera	16(5)	5(5)	75(5)	15(5)	10(10)	4(3)	10(10)	15(15)	25(15)	0	15(10)	0
Corrodentia	0	0	0	0	0	0	0	0	75(45)	5(5)	6(5)	4(2)
Thysanoptera	0	0	0	0	0	36(15)	0	0	0	0	6(5)	0
Coleoptera larvae	70(30)	15(15)	26(5)	16(5)	446(95)	12(2)	276(65)	120(40)	110(30)	129(40)	87(25)	65(6)
Coleoptera adult	10(10)	35(15)	20(10)	14(6)	20(5)	25(5)	10(10)	35(15)	20(10)	9(5)	64(6)	20(3)
Lepidoptera larvae	5(5)	0	10(10)	0	0	16(6)	6(5)	0	35(15)	0	10(10)	4(3)
Diptera larvae	70(40)	30(10)	176(55)	62(2)	15(5)	112(20)	109(30)	59(20)	252(6)	50(20)	55(25)	124(45)
Hymenoptera	130(50)	960(150)	20(10)	45(15)	855(165)	7(6)	380(180)	410(15)	60(20)	167(55)	166(45)	27(15)

**Figure 2:**
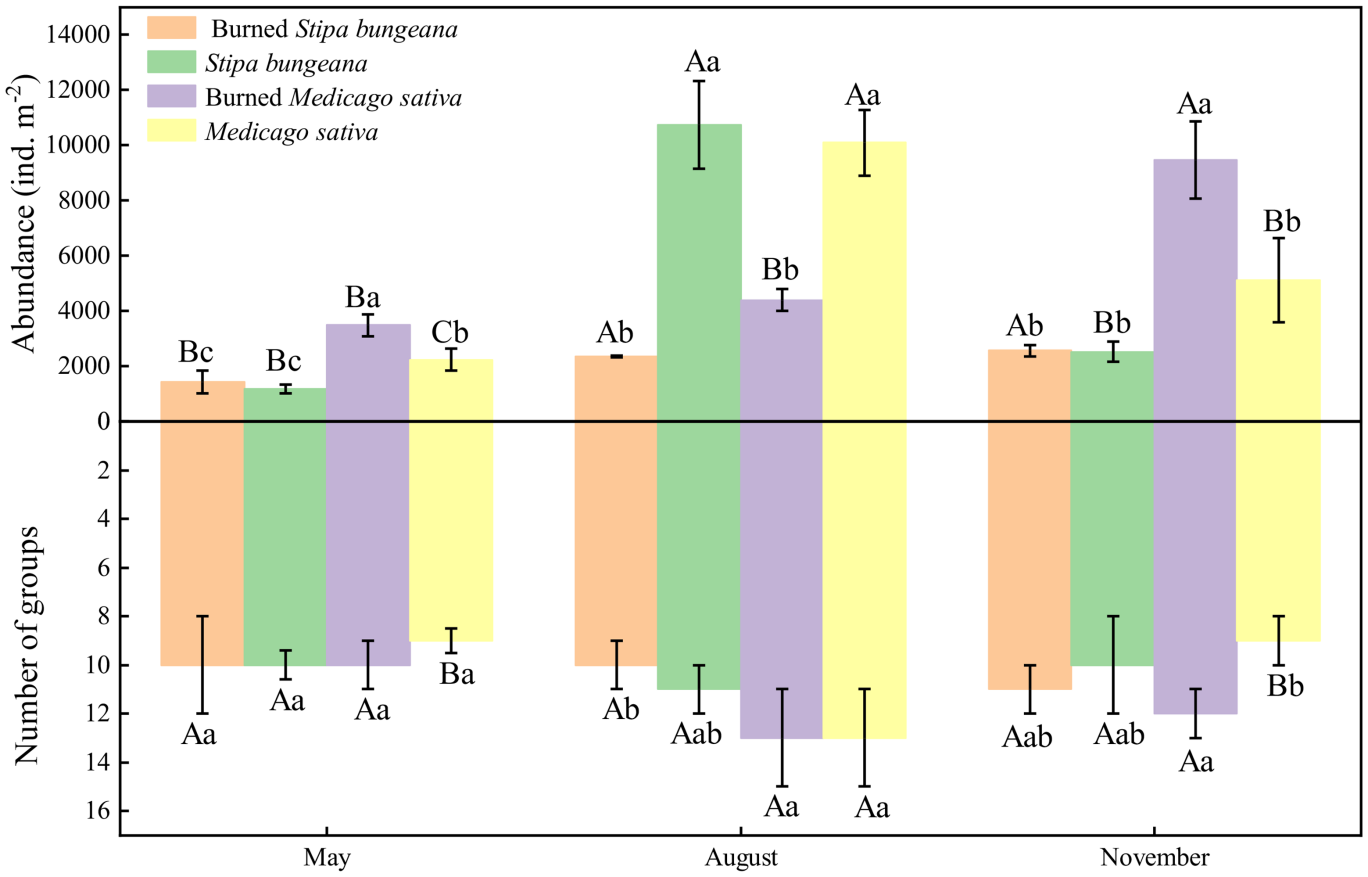
Dynamics of soil arthropods abundance and group numbers in *Stipa bungeana*, Burned *Stipa bungeana*, *Medicago sativa*, and Burned *Medicago sativa* habitats. Different lowercase letters indicate significant difference among different habitat types at the same sampling period (*p* < 0.05, Duncan’s test). Different uppercase letters indicate significant difference among different sampling periods at the same habitat (*p* < 0.05, Duncan’s test).

**Figure 3:**
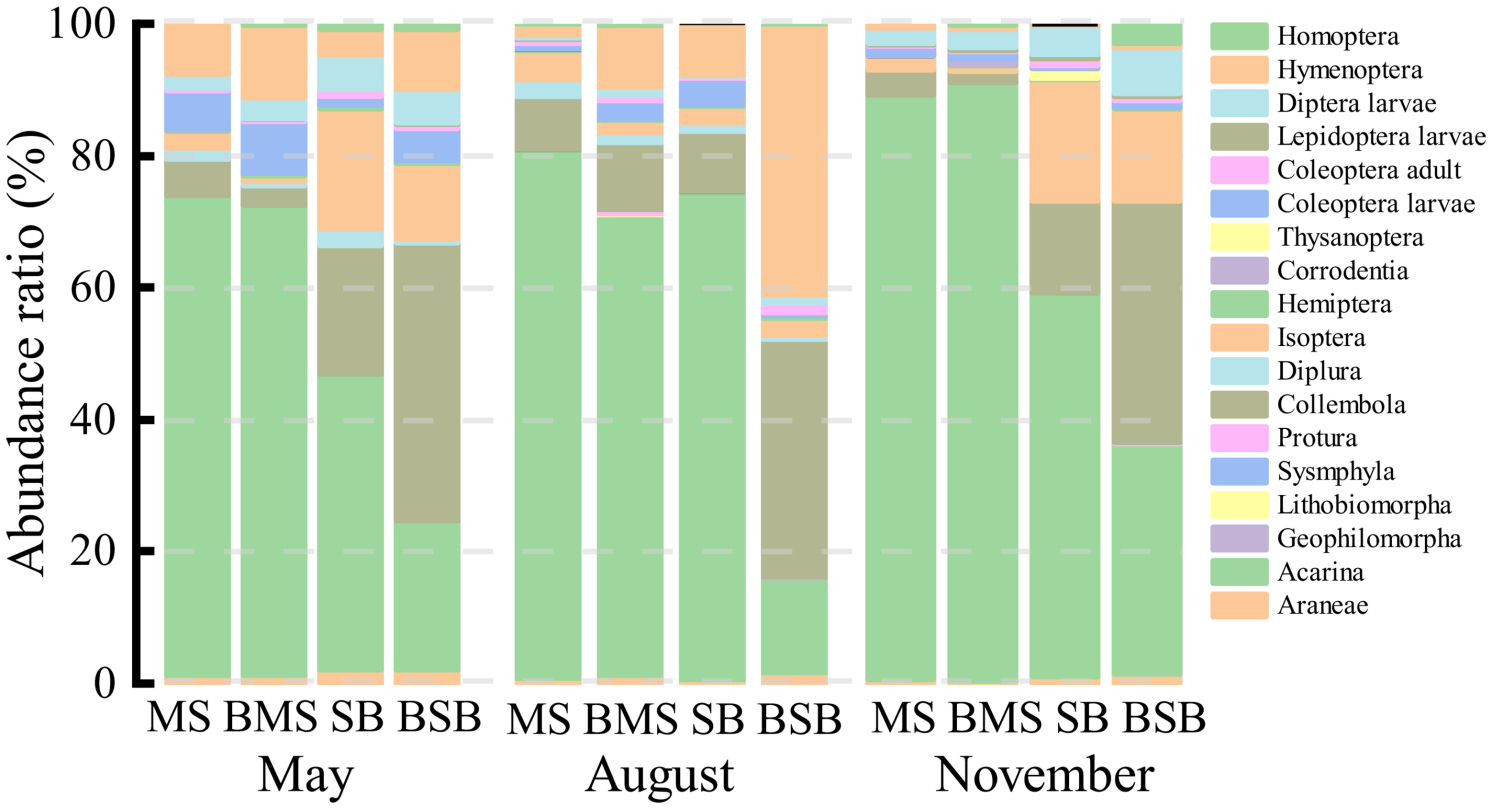
The proportion of a certain group abundance to the total group abundance in May, August, and November. SB, *Stipa bungeana* habitat; BSB, Burned *Stipa bungeana* habitat; MS, *Medicago sativa* habitat; BMS, Burned *Medicago sativa* habitat.

The soil arthropod abundance in the unburned (*S. bungeana* and *M. sativa*) habitats showed a trend of increasing first and then decreasing during the three sampling periods and was significantly (*p* < 0.05) greater in August than in May and November. In addition, the number of soil arthropod groups also showed a trend of first increasing and then decreasing. In contrast to the unburned habitat, the abundance of soil arthropods in the burned *S. bungeana* and Burned *M. sativa* habitats gradually increased during the three sampling periods (Figure 2). For the entire area, soil arthropod abundance was significantly affected by vegetation type (S, *p* < 0.001), wildfire (W, *p* < 0.01), and sampling time (T, *p* < 0.001), but the number of arthropod groups was only significantly affected by sampling time (T, *p* < 0.01; Table 2). In addition, the community composition of soil arthropods was different between SB and MS. The effects of fire on soil arthropod community composition in the SB habitat were greater than those in the MS habitat (Figure 3).

**Table 2:** ANOVA results for environmental factors and main groups as affected by habitat types (S), wildfire (W), sampling time (T) and their interactions (S × W, S × T, W × T, S × W × T).

Parameter	Value of *p*						
	S	W	T	S × W	S × T	W × T	S × W × T
Acari	<0.001^***^	0.005^**^	<0.001^***^	0.006^**^	0.002^**^	<0.001^***^	0.295
Collembola	<0.001^***^	0.127	<0.001^***^	<0.001^***^	0.161	<0.001^***^	0.197
Diplura	0.003^**^	<0.001^***^	<0.001^***^	0.216	0.002^**^	<0.001^***^	0.262
Isoptera	<0.001^***^	<0.001^***^	0.004^**^	0.813	<0.001^***^	0.004^**^	0.330
Coleoptera larvae	0.018^*^	0.101	<0.001^***^	<0.001^***^	<0.001^***^	<0.001^***^	<0.001^***^
Diptera larvae	0.009^**^	<0.001^***^	<0.001^***^	0.144	0.567	0.013^*^	0.389
Hymenoptera	<0.001^***^	0.001^**^	<0.001^***^	0.158	<0.001^***^	0.144	0.720
Abundance	<0.001^***^	0.007^**^	<0.001^***^	0.008^**^	0.004^**^	<0.001^***^	0.352
Number of groups	0.074	0.598	0.008^**^	0.013^*^	0.013^*^	0.092	0.613
SBD	0.039^*^	0.981	0.197	0.518	0.748	0.504	0.339
SWC	0.027^*^	0.008^**^	<0.001^***^	0.165	0.001^**^	<0.001^***^	0.771
pH	0.040^*^	0.059	0.053	0.011^*^	0.590	0.003^**^	0.067
SOC	0.138	0.923	0.014^*^	0.317	0.944	0.208	0.019^*^
TN	0.025^*^	0.950	0.858	0.625	0.129	0.610	0.813
TP	0.239	0.002^**^	0.082	0.162	0.245	0.283	0.231
AN	0.036^*^	0.76	<0.001^***^	0.843	0.479	0.010^*^	0.262
NN	<0.001^***^	0.010^*^	0.704	0.016^*^	0.414	0.659	0.462
AP	0.036^*^	0.409	<0.001^***^	0.462	0.560	0.775	0.322
VC	<0.001^***^	<0.001^***^	<0.001^***^	0.235	0.564	<0.001^***^	0.021^*^
LT	<0.001^***^	<0.001^***^	<0.001^***^	<0.001^***^	0.199	0.604	0.886

SWC, Soil water content; SBD, Soil bulk density; pH, Soil pH; SOC, Soil organic carbon; TN, Soil total nitrogen; TP, Soil total phosphorus; AP, Available phosphorus; AN, Ammonia nitrogen; NN, Nitrate nitrogen; VC, Vegetation coverage; LT, Litter thickness.

* *p* < 0.05;

** *p* < 0.01;

*** *p* < 0.001.

### Response of soil arthropods community to different habitats and time

**Figure 4:**
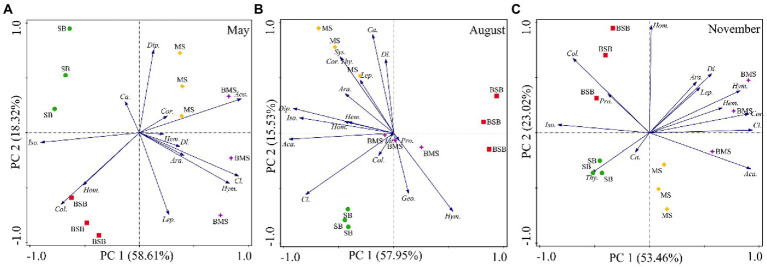
Principal component analysis of soil arthropods during May **(A)**, August **(B)**, and November **(C)** with the habitats as an overlay. Ara., Araneae; Aca., Acari; Geo., Geophilomorpha; Lit., Lithobiomorpha; Sys., Sysmphyla; Pro., Protura; Col., Collembola; Dip., Diplura; Iso., Isoptera; Hem., Hemiptera; Cor., Corrodentia; Thy., Thysanoptera; Cl., Coleoptera larvae; *Ca.,* Coleoptera adult; Lep., Lepidoptera larvae; Dl., Diptera larvae; Hym., Hymenoptera; Hom., Homoptera; SB, *Stipa bungeana* habitat; BSB, Burned *Stipa bungeana* habitat; MS, *Medicago sativa* habitat; BMS, Burned *Medicago sativa* habitat.

Soil arthropod communities differed among the four habitats in August, especially in May and November (Figure 4). The main groups showing significant differences among communities varied with sampling time. The groups affecting the distribution on the PC1 axis in May were mainly Acari and Isoptera, and those affecting the distribution on the PC2 axis were Diplura and Lepidoptera larvae. The main groups associated with the separation of the PC1 axis were Acari and Diplura in August, and Acari, Coleoptera larvae, and Isoptera in November. The main groups associated with the separation of the PC2 axis were adult Coleoptera and Diptera larvae in August and Homoptera in November. Overall, the community structure of soil arthropods differed among habitats. Most groups preferred to occur in MS and BMS habitats.

**Figure 5:**
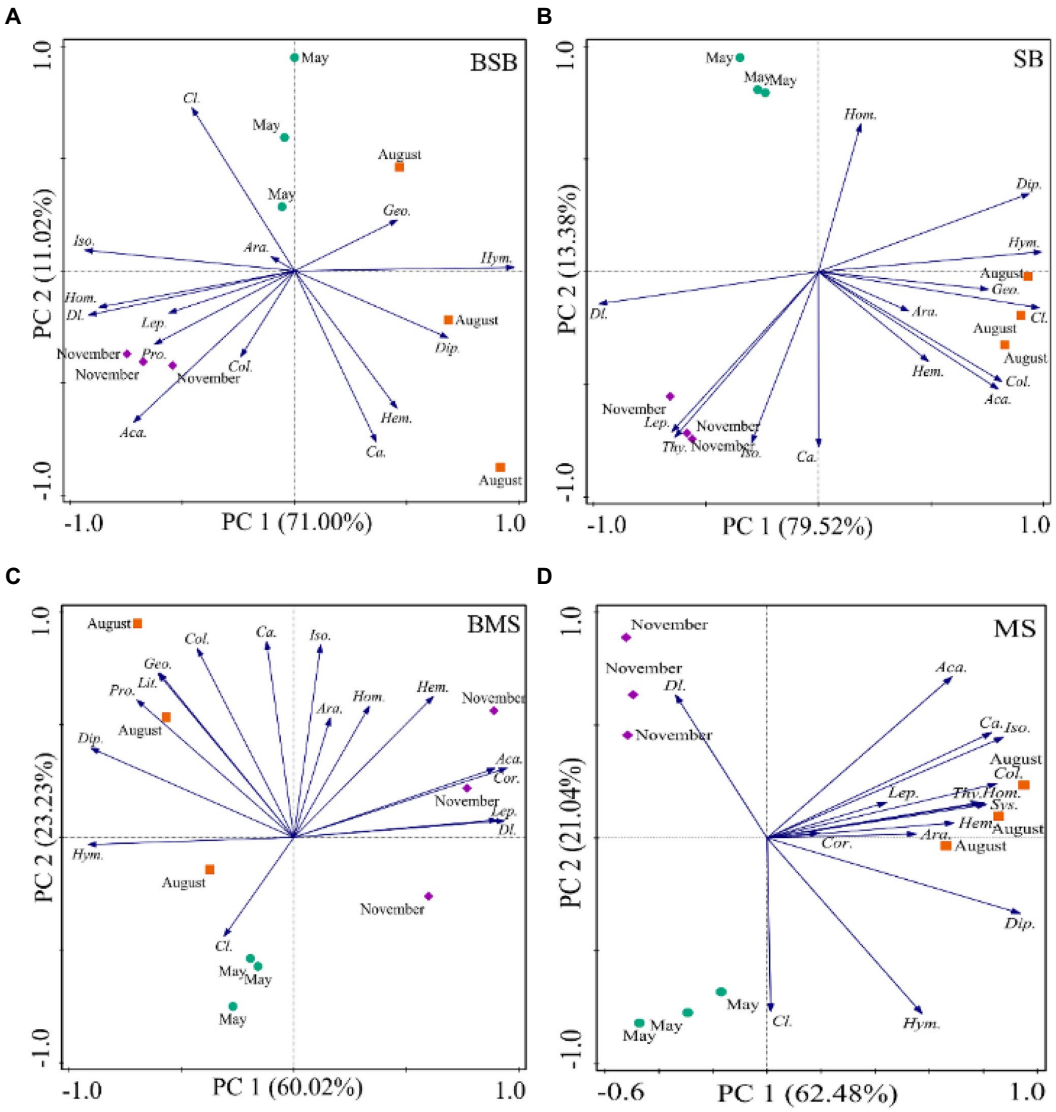
Principal component analyses of the temporal dynamics in the community structure of the soil arthropods in BSB **(A)**, SB **(B)**, BMS **(C)**, and MS **(D)**. Ara., Araneae; Aca., Acari; Geo., Geophilomorpha; Lit., Lithobiomorpha; Sys., Sysmphyla; Pro., Protura; Col., Collembola; Dip., Diplura; Iso., Isoptera; Hem., Hemiptera; Cor., Corrodentia; Thy., Thysanoptera; Cl., Coleoptera larvae; *Ca.,* Coleoptera adult; Lep., Lepidoptera larvae; Dl., Diptera larvae; Hym., Hymenoptera; Hom., Homoptera; SB, *Stipa bungeana* habitat; BSB, Burned *Stipa bungeana* habitat; MS, *Medicago sativa* habitat; BMS, Burned *Medicago sativa* habitat.

Considering the seasonal dynamics of soil arthropods, PCA results showed a similar community composition across all sampling periods. The first two axes explained 82.02%, 92.90%, 83.25%, and 83.52% of total variation in BSB, SB, BMS, and MS, respectively (Figure 5). Soil arthropods in the SB habitat were separated from one another on the PC1 and PC2 axes in May, August, and November, but not in the BSB, BMS, or MS habitats. Moreover, the main groups that determined the differences among soil arthropods varied among the habitats. The main groups related to the PC1 axis were Hymenoptera and Isoptera in the BSB habitat; Hymenoptera, Coleoptera larvae, and Diptera larvae in the SB habitat; Diptera larvae and Hymenoptera in the BMS habitat; and Diplura and Isoptera in the MS habitat. The main groups related to the separation along the PC2 axis were Coleoptera larvae and Coleoptera adults in the BSB habitat; Homoptera and Coleoptera adults in the SB habitat; Coleoptera adult and Isoptera in the BMS habitat; and Coleoptera larvae and Diptera larvae in the MS habitat. Overall, the distribution of soil arthropods in the BSB and BMS habitats was concentrated in August and November, whereas that in the SB and MS habitats was concentrated in August.

### Response of soil/vegetation parameters to habitats, wildfire, and time

**Figure 6:**
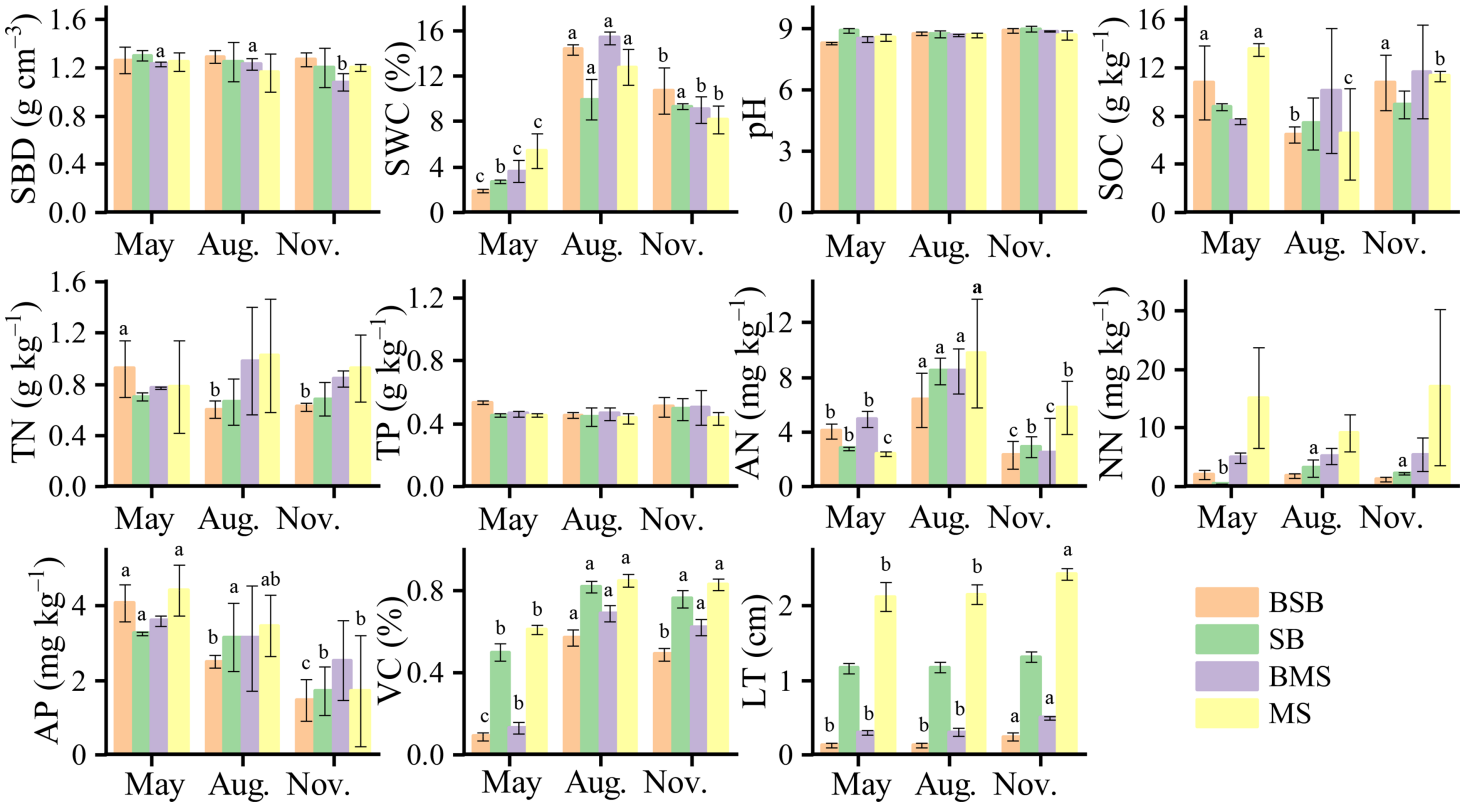
Properties of soil and vegetation in *Stipa bungeana* habitat (SB), Burned *Stipa bungeana* habitat (BSB), *Medicago sativa* habitat (MS), and Burned *Medicago sativa* habitat (BMS). Different lowercase letters denote significant differences among different sampling times (ANOVA with LSD test, *p* < 0.05). SWC, Soil water content; SBD, Soil bulk density; pH, Soil pH; SOC, Soil organic carbon; TN, Soil total nitrogen; TP, Soil total phosphorus; AP, Available phosphorus; AN, Ammonia nitrogen; NN, Nitrate nitrogen; VC, Vegetation coverage; LT, Litter thickness.

All parameters, except TP, were significantly (*p* < 0.05) affected by habitat type (Table 2). Most parameters (SWC, SOC, TN, VC, and LT) were higher in the *M. sativa* habitat than in the *S. bungeana* habitat (Figure 6). Except for pH and TP, all soil properties were significantly (*p* < 0.05) affected by sampling time. SWC, TP, NN, LT, and VC were significantly (*p* < 0.05) affected by the wildfire. The unburned habitats had a greater SWC in May than the burned habitats, but the opposite trend was observed in August and November. The AN for all habitats was maximum in August, and the AP had a maximum in May. LT and VC were greater in the unburned habitats and gradually increased with time.

### Effects of environment factors on soil arthropods

**Figure 7:**
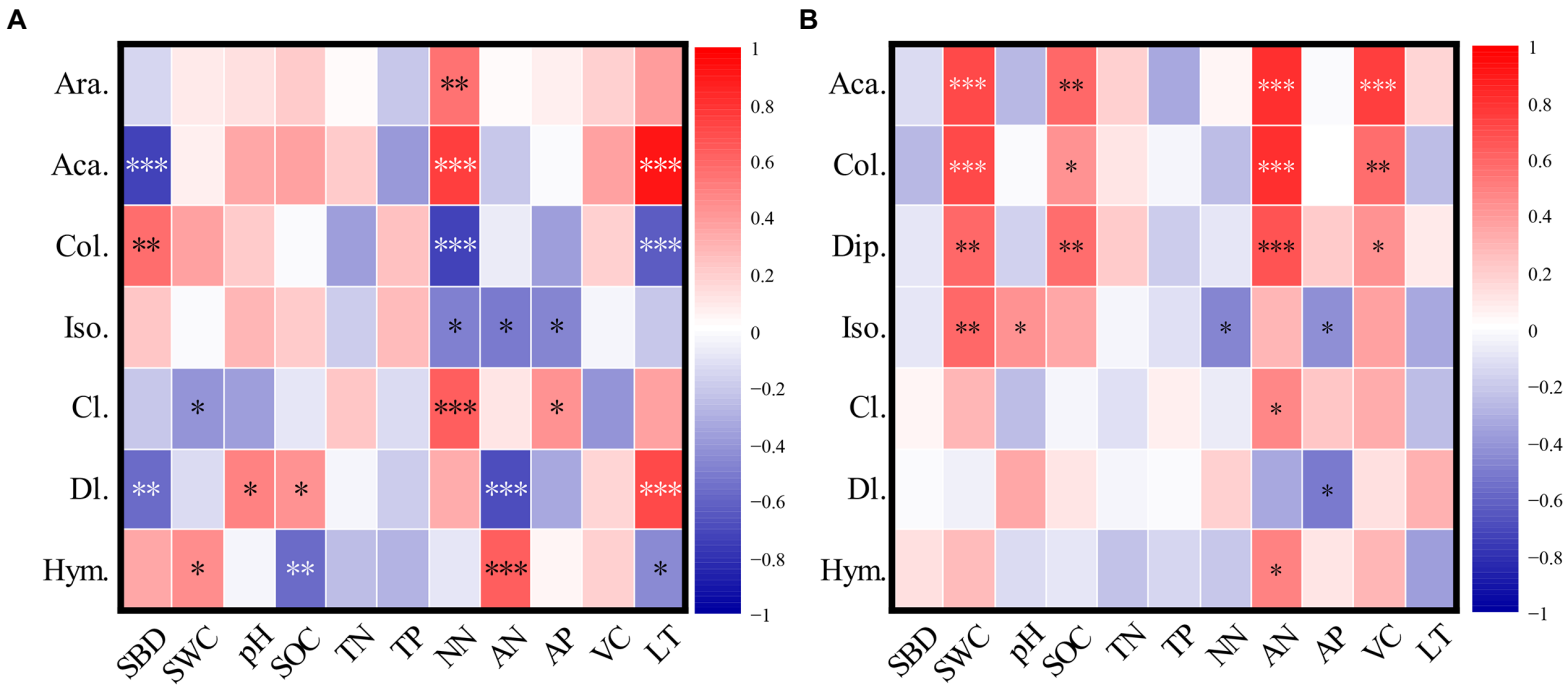
Pearson correlation coefficients between main groups of soil arthropods and environmental factors (**A** is the burned habitats; **B** is the unburned habitats). Ara., Araneae; Aca., Acari; Col., Collembola; Dip., Diplura; Iso., Isoptera; Cl., Coleoptera larvae; Dl., Diptera larvae; Hym., Hymenoptera; SWC, Soil water content; SBD, Soil bulk density; pH, Soil pH; SOC, Soil organic carbon (SOC); TN, Soil total nitrogen; TP, Soil total phosphorus; AP, Available phosphorus; AN, Ammonia nitrogen; NN, Nitrate nitrogen; VC, Vegetation coverage; LT: Litter thickness. ^*^p < 0.05; ^**^p < 0.01; ^***^p < 0.001.

Araneae was a common group in burned habitats and was significantly (*p* < 0.05) positively correlated with NN (Figure 7). Diplura was a common group of unburned habitats, and was positively (*p* < 0.05) correlated with SWC, SOC, AN, and VC. Acari was positively correlated (*p* < 0.05) with NN and LT, but negatively correlated with SBD in burned habitats (*p* > 0.05). However, Collembola showed opposite trends in burned habitats. Acari and Collembola showed similar trends in unburned habitats. They were positively correlated (*p* > 0.05) with SWC, SOC, AN, and VC. The environmental factors significantly associated with Diptera larvae and Hymenoptera were greater in burned habitats than in unburned habitats.

**Figure 8:**
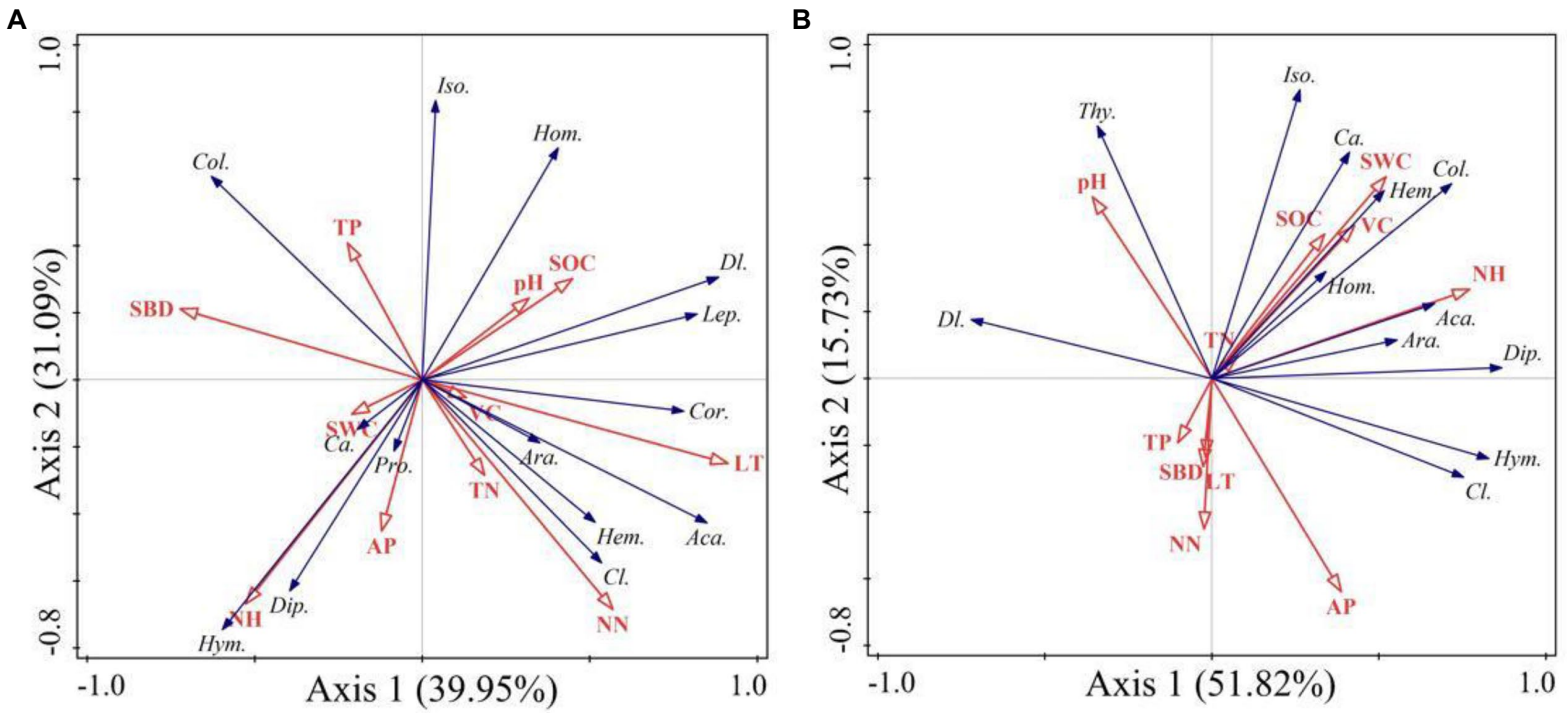
Redundancy analysis (RDA) showing the relationship between soil arthropods composition and environment factors (**A** is the burned habitats; **B** is the unburned habitats). Ara., Araneae; Aca., Acari; Pro., Protura; Col., Collembola; Dip., Diplura; Iso., Isoptera; Hem., Hemiptera; Thy., Thysanoptera; Cor., Corrodentia; Cl., Coleoptera larvae; *Ca.*, Coleoptera adult; Lep., Lepidoptera larvae; Dl., Diptera larvae; Hym., Hymenoptera; Hom., Homoptera.

**Figure 9:**
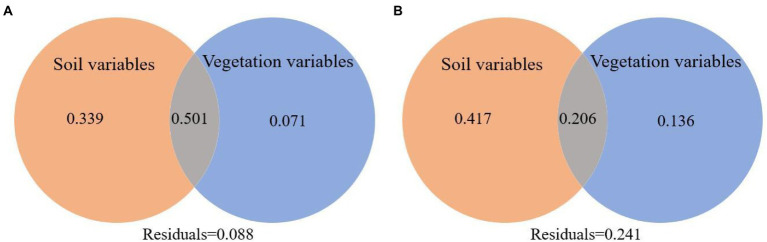
Variation partitioning of total variation in overall arthropods data in terms of the pure effect of the soil variables and vegetation variables and the joint effect of all variables considered (**A** is the burned habitats; **B** is the unburned habitats).

According to the RDA analysis, the first two axes of burned and unburned habitats explained 71.04% and 67.55% of variation in soil arthropods, respectively (Figure 8). Simple term effects showed that the distribution of soil arthropods was greatly affected by SBD, SOC, NN, AN, AP, and LT in burned habitats (Table 3). Moreover, forward selection of environmental factor variables found that the LT and AN statistically explained 35.60% and 23.30% of the variations, respectively. In contrast, soil arthropods in unburned habitats were mainly affected by SWC, AN, AP, and VC. Moreover, forward selection of environmental factor variables found that the AN and VC statistically explained 33.10% and 16.50% of the variations, respectively. The distribution of soil arthropods is affected by the individual and interaction effects of these environmental factors. Variation partitioning showed that the largest fraction of explained variation was accounted for by a joint effect (50.10%) in the burned habitats, followed by pure soil variables (39.90%; Figure 9). In the unburned habitats, soil variables explained 62.30% of the variation in overall arthropods, but of this, only 41.70% was unique to this predictor, with the remaining 20.60% also explained by a joint effect.

**Table 3:** Simple term effects and forward selection of environmental variables based on Monte Carlo permutation tests from the redundancy analysis (RDA).

Variable	Burned habitats				Unburned habitats			
	Simple term effects		Forward section		Simple term effects		Forward section	
	Explains (%)	*p*	Explains (%)	*p*	Explains (%)	*p*	Explains (%)	*p*
SBD	22.800	0.002	3.000	0.002	2.600	0.626	6.200	0.022
SWC	8.400	0.084	1.200	0.114	22.500	0.008	2.900	0.150
pH	10.100	0.048	1.800	0.026	12.700	0.042	2.400	0.174
SOC	11.700	0.020	4.500	0.018	11.500	0.052	3.300	0.106
TN	5.300	0.312	3.300	0.006	1.700	0.800	1.800	0.238
TP	8.800	0.096	0.300	0.796	1.700	0.796	1.600	0.256
NN	28.000	0.002	2.900	0.112	6.600	0.214	2.500	0.104
AN	26.400	0.002	23.300	0.002	33.100	0.002	33.100	0.002
AP	8.800	0.070	6.400	0.002	14.800	0.016	7.500	0.034
VC	7.200	0.144	9.900	0.002	18.600	0.010	16.500	0.014
LT	35.600	0.002	35.600	0.002	6.100	0.192	2.600	0.158

SWC, Soil water content; SBD, Soil bulk density; pH, Soil pH; SOC, Soil organic carbon; TN, Soil total nitrogen; TP, Soil total phosphorus; AP, Available phosphorus; AN, Ammonia nitrogen; NN, Nitrate nitrogen; VC, Vegetation coverage, LT, Litter thickness.

## Discussion

Changes in fire activity threaten habitat biodiversity worldwide (Kelly et al., 2020). Fire reduces the taxonomic diversity of soil macrofauna and severely affects the soil faunal community (Gongalsky and Persson, 2013). However, the results were not completely in line with those of previous studies and our hypothesis. This study showed that fire significantly reduced soil arthropod abundance but had no significant effect on the number of soil arthropod groups (Figure 2; Table 2). In general, fire events affect soil fauna communities both directly (by killing or injuring organisms) and indirectly (by reducing habitat availability and food resources; Certini, 2005; Coyle et al., 2017). In addition, the effects of fire on soil biological properties have a great relationship with the situation of the fire itself (residence time, heterogeneity, and severity) and environmental factors (topography, season, soil moisture, and weather conditions; Bellido, 1987; Hellberg et al., 2004; Lavoie and Mack, 2012). Our results may be attributed to the fact that the fire event was the first fire in the area and occurred in winter. In winter, most soil fauna enter the ground or the bottom of the humus layer to escape low temperatures. Moreover, fire is classified as light. These characteristics indicate that the direct lethal effect of fire on soil arthropods is weak. In addition, there was no significant difference in soil arthropod abundance at the beginning of the fire (May), but this difference gradually increased over time (Figure 2). A possible reason for this is that fire altered habitat characteristics (Figure 6) and thus restricted the survival and reproduction of soil arthropods. Therefore, our first hypothesis needs to be changed to suggest that light fire had a greater effect on soil arthropod abundance than the groups. This effect was mainly attributed to changes in habitat characteristics.

In this study, we also found that soil arthropod abundance in unburned habitats (SB and MS) first increased and then decreased with sampling time, with the highest value observed in August (Figure 2). This finding is associated with temporal variations in environmental factors (Yin et al., 2018). Common environmental factors, such as soil properties, can provide food and habitat and change the composition of the soil fauna (Birkhofer et al., 2012; Carmen et al., 2013). The seasonal dynamics of plant properties may directly (microclimate and resource availability) or indirectly (soil properties) affect soil fauna communities (Irmler, 2006). In this study, soil properties (e.g., SWC, AN, and SOC) and vegetation properties (e.g., LT and VC) were significantly affected by the sampling time (Figure 6; Table 2). In addition, Lindberg (2003), Fu et al. (2009), and Yin et al. (2010) reported that changes in temperature and precipitation accompanied by seasonal changes also affect soil fauna. Many studies have confirmed that soil fauna exhibit significant seasonal variations (Pen-Mouratov et al., 2004; Wiwatwitaya and Takeda, 2005; Hugo-Coetzee and Avenant, 2011). Moreover, we found that temperature and precipitation were significantly positively correlated with most soil arthropods (Supplementary Figure S2B). The reason for the high concentration of soil arthropods in unburned habitats in August may be that high temperatures and precipitation in summer directly promote the survival and reproduction of soil arthropods and indirectly enhance the growth of vegetation to create suitable habitats and food sources for soil arthropods.

Interestingly, we found that the abundance of soil arthropods in burned habitats (BSB and BMS) increased with sampling time (Figure 2). However, this phenomenon was inconsistent with the unburned habitats. In general, the post-fire recovery of soil faunal communities depends on abiotic and organism traits (Gongalsky et al., 2012). Compared with organism traits, changes in abiotic factors (habitat characteristics) caused by fire were the main factors leading to differences in soil arthropods. A possible reason is that fire destroys the preferred part of the soil habitat for most soil organisms, that is, the litter and uppermost humus layer, or if the fire is very severe, the entire humus layer (Gongalsky and Persson, 2013). Additionally, fire-induced changes in plant communities may lead to variations in the microclimate, resource availability, and soil properties among different habitats, directly or indirectly affecting the soil arthropod community (Irmler, 2006). Unburned habitats are stable systems in which soil arthropods are greatly affected by seasonal changes in climate (e.g., temperature and precipitation; Zhu et al., 2005). In contrast, soil and vegetation properties closely related to soil arthropods in burned habitats have also been gradually restored (Bandyopadhyaya et al., 2002; Sechi et al., 2014). However, environmental factors (e.g., temperature and precipitation) had less impact on the restoration of soil arthropods in burned habitats than the soil arthropods themselves. In the early stage of restoration, competition between soil arthropods was small, and predators had not yet appeared; hence, soil arthropods were able to reach full development and reproduction, and their abundance gradually increased. Unexpectedly, the number of arthropod groups did not increase significantly with recovery time. Therefore, the second hypothesis was modified.

Taken together, soil fauna communities are closely related to environmental factors (Bardgett and van der Putten, 2014; Yin et al., 2018). It has been proven that the distribution of soil fauna is affected by vegetation and soil quality (Coulson et al., 2003; Viketoft, 2007; Yan et al., 2012). Nevertheless, the environmental factors driving the changes in soil arthropods were different in burned and unburned habitats (Figure 8). Similar results have been reported by Gorbunova et al. (2017). It is generally accepted that habitats with litter or humus layers have more evident surface aggregation of soil fauna (Wu et al., 2006; Huang et al., 2010). However, after the fire, the understory vegetation and litter almost completely disappeared, and the habitat and food sources of soil arthropods were severely damaged (Yang et al., 2013). The living environment of soil fauna is improved by the accumulation of these substances, especially litter. This might explain the significant positive effects of LT on soil arthropods. In unburned habitats, VC showed significant seasonal changes, which were highly consistent with most soil arthropod changes (Figure 6). VC reflects the growth status of the plants. A high VC can block solar radiation and light (Zhu et al., 2021), provide sufficient food sources, and therefore may be beneficial to the survival of soil arthropods, especially Acari, Diplura, and Collembola. Previous studies have shown that the type, diversity, and composition of vegetation significantly affect the survival of soil fauna, and its complexity level is positively correlated with soil fauna diversity (Lin et al., 2009; Huang et al., 2010). These results indicate that both soil and vegetation reduced the rate of interpretation of arthropod variation after fire. Therefore, the hypothesis that vegetation alone is responsible for the short-term recovery dynamics of soil arthropods following a fire is insufficient. Fire is an important natural disturbance factor with dual attributes (Ponomarev et al., 2016), and its influence on the development of species diversity in time and space is one of the most important factors for understanding the global ecosystem distribution and species diversity (Pausas and Ribeiro, 2017). Thus, in-depth research on the changes in environmental factors caused by fire must be conducted to evaluate the integrated changes in the soil fauna after fire.

## Conclusion

We concluded that light fires in winter had a greater impact on soil arthropod numbers than the groups. Soil arthropods in unburned habitats had significant seasonal dynamics, and in burned habitats, they gradually increased and exceeded the unburned habitats. Moreover, our findings revealed that fire-induced changes in soil and vegetation properties (e.g., AN, LT, and VC) are major determinants of soil arthropod recovery dynamics. The results provide strong evidence that the effects of fire on soil fauna are dependent on habitat changes. This study reveals the short-term recovery dynamics of soil arthropods after a fire and provides references for post-fire ecological restoration and biodiversity protection.

## Data availability statement

The raw data supporting the conclusions of this article will be made available by the authors, without undue reservation.

## Ethics statement

Ethical review and approval was not required for the animal study because There are no ethical issues involved in this study.

## Author contributions

XY, R-TL, M-AS, X-RW, and T-CL contributed to the conception and design of the study. M-YC, Z-YL, XY, and MG performed the experiments. XY wrote the manuscript. T-CL made a great contribution in the process of revising the manuscript. Y-CD provided funds and revised the manuscript. All authors contributed to the article and approved the submitted version.

## Funding

This research was supported by the Open Fund for Key Lab. of Ecological Study of Ningxia University (Grant number: LDER2022Z01), Chinese Universities Scientific Fund (2452022335) and the State Key Laboratory of Soil Erosion and Dryland Farming on the Loess Plateau (A314021402-2021012).

## Conflict of interest

The authors declare that the research was conducted in the absence of any commercial or financial relationships that could be construed as a potential conflict of interest.

## Publisher’s note

All claims expressed in this article are solely those of the authors and do not necessarily represent those of their affiliated organizations, or those of the publisher, the editors and the reviewers. Any product that may be evaluated in this article, or claim that may be made by its manufacturer, is not guaranteed or endorsed by the publisher.
