# Supplementary material for: Short-term effects of wildfire on soil arthropods in a semi-arid grassland on the Loess Plateau
Source: Front Microbiol. 2022 Oct 21;13:989351. doi: 10.3389/fmicb.2022.989351 (PMC9634180; doi:10.3389/fmicb.2022.989351)
Supplement: Supplementary file 1 [file Data_Sheet_1.docx]

**electronic supplementary material**

**Fig. S1.** Monthly mean temperature (℃) and precipitation (mm) in the study area, during 2009-2018.

**Fig. S2.** Pearson correlation coefficients between temperature (℃) and precipitation (mm) with environmental factors (a is the burned habitats; b is the unburned habitats).

**Abbreviations:** Araneae (Ara.), Acari (Aca.), Collembola (Col.), Diplura (Dip.), Isoptera (Iso.), Coleoptera larvae (Cl.), Diptera larvae (Dl.), Hymenoptera (Hym.), Soil water content (SWC), Soil bulk density (SBD), Soil pH (pH), Soil organic carbon (SOC), Soil total nitrogen (TN), Soil total phosphorus (TP), Available phosphorus (AP), Ammonia nitrogen (AN), Nitrate nitrogen (NN), Vegetation coverage (VC), Litter thickness (LT), Temperature (TEM), and Precipitation (PRE). *p<0.05; **p<0.01; ***p<0.001.
